# Biotechnological production of 1,2,4-butanetriol: An efficient process to synthesize energetic material precursor from renewable biomass

**DOI:** 10.1038/srep18149

**Published:** 2015-12-16

**Authors:** Yujin Cao, Wei Niu, Jiantao Guo, Mo Xian, Huizhou Liu

**Affiliations:** 1 CAS Key Laboratory of Biobased Materials, Qingdao Institute of Bioenergy and Bioprocess Technology, Chinese Academy of Sciences, Qingdao 266101, China; 2 Department of Chemistry, University of Nebraska-Lincoln, Lincoln, Nebraska 68588, United States

## Abstract

1,2,4-Butanetriol (BT) is a valuable chemical with extensive applications in many different fields. The traditional chemical routes to synthesize BT suffer from many drawbacks, e.g., harsh reaction conditions, multiple steps and poor selectivity, limiting its industrial production. In this study, an engineered *Escherichia coli* strain was constructed to produce BT from xylose, which is a major component of the lignocellulosic biomass. Through the coexpression of a xylose dehydrogenase (*CCxylB*) and a xylonolactonase (*xylC*) from *Caulobacter crescentus*, native *E. coli* xylonate dehydratase (*yjhG*), a 2-keto acid decarboxylase from *Pseudomonas putida* (*mdlC*) and native *E. coli* aldehyde reductase (*adhP*) in *E. coli* BL21 star(DE3), the recombinant strain could efficiently convert xylose to BT. Furthermore, the competitive pathway responsible for xylose metabolism in *E. coli* was blocked by disrupting two genes (*xylA* and *EcxylB*) encoding xylose isomerase and xyloluse kinase. Under fed-batch conditions, the engineered strain BL21ΔxylAB/pE-mdlCxylBC&pA-adhPyjhG produced up to 3.92 g/L of BT from 20 g/L of xylose, corresponding to a molar yield of 27.7%. These results suggest that the engineered *E. coli* has a promising prospect for the large-scale production of BT.

1,2,4-Butanetriol (BT) is a four-carbon polyol with three hydrophilic hydroxyl groups. As an important fine chemical, BT has versatile applications in many different fields and attracted considerable interest in the past few years. For instance, BT can be used for making polyurethane foams. These foams have the same compression-bending characteristics as natural rubber^1^. BT severs as the recording agent of high-quality inks and renders the inks having well balanced anti-feathering and penetrability effects^2^. Optically active BT is also a potential building block for the synthesis of various pharmaceuticals, such as Crestor and Zetia^3^. Finally, BT is the direct precursor for the manufacture of butanetriol trinitrate, an excellent energetic plasticizer to replace nitroglycerin, which is less volatile, thermally more stable and offers better low-temperature properties^4^,^5^.

BT is traditionally manufactured through chemical routes using glycidol^6^, 2-butene-1,4-diol^7^, 3,4-dihydroxybutanoate^8^ or malate^9^,^10^ as the starting materials. Among them, the high pressure catalytic hydrogenation of esterified malate is currently commercially available. However, this process requires NaBH_4_ as the reducing agent. For each ton of BT to be synthesized, multiple tons of borate salts are generated as the by-products, thus resulting in high production costs and severe environmental pollution^11^. In addition, all of the chemical synthetic routes suffer from similar drawbacks, e.g., harsh reaction conditions, multiple steps and poor selectivity. These difficulties create hurdles that hamper further applications of the petrochemical-based BT.

In recent years, biomass utilization for the production of value-added chemicals is attracting considerable interest^12^,^13^. Xylose is the second most abundant sugar in nature and a major constituent of hemicellulose in lignocellulosic biomass^14^. A number of wild-type microbial strains or metabolically engineered strains have been isolated or constructed to ferment xylose for the production of ethanol^15^, xylitol^16^, succinate^17^, lactate^18^ and other important products. The utilization of biomass-derived xylose to produce bio-based chemicals offers many advantages over the traditional petrochemical routes in that it is made from renewable resources, possesses mild transformation conditions and reduces the environmental pollution. Researchers also explored new routes for BT production and finally they established a new process to produce BT from xylose by microbial conversion^19^. Through the introduction of a 2-keto acid decarboxylase (*mdlC*) from *Pseudomonas putita*^20^ to *Escherichia coli*, the recombinant strain could efficiently covert xylonate to BT. Meanwhile, the feedstock xylonate could be obtained by microbial oxidation of xylose by *Pseudomonas fragi*^21^. The entire metabolic pathway from xylose to BT is proposed in Fig. 1. Although many wild-type bacterial species can produce xylonate in high yields^21^,^22^,^23^, these natural producers always require expensive nutrient media and take several days to reach the maximum titer. Meanwhile, separation and purification of xylonate from the fermentation broth is also a difficult task. As is known to all, the downstream processing of organic acids is estimated to make up the major cost in its microbial production^24^. Therefore, the two-step fermentation process would be economically unfeasible for large-scale production. A one-pot conversion from xylose to BT would be more reliable for industrial applications.

The construction of a direct BT-producing *E. coli* strain from xylose was presented in this study. The *Caulobacter crescentus* xylose dehydrogenase (*CCxylB*), xylonolactonase (*xylC*) and *P. putita mdlC* were coexpressed with native *yjhG* (encoding xylonate dehydratase) and *adhP* (encoding aldehyde reductase/alcohol dehydrogenase) in *E. coli* BL21 star(DE3). To avoid the consumption of xylose for cell growth, the endogenous xylose catabolic pathway was blocked by disrupting two genes, *xylA* (encoding xylose isomerase) and *EcxylB* (encoding xylulose kinase) responsible for xylose utilization. The engineered strain was cultured under fed-batch conditions to evaluate the potential for large-scale BT production.

## Results

### Identification of recombinant enzymes expressed in E. coli

*E. coli* BL21 star(DE3) was chosen as the host for recombinant proteins expression and BT production in this work. This strain has a genotype that promotes mRNA stability and protein yield and thus is ideal for use with low copy number, T7 promoter-based plasmids. In order to express the CCXylB, XylC, YjhG, MdlC and AdhP enzymes which constitute the entire metabolic pathway from xylose to BT, we cloned the coding regions of these genes into the expression vectors pET28a, pTrcHis2B, pETDuet-1 and pACYCDuet-1, respectively. The expression constructs were checked by restriction enzyme digestion and DNA sequencing. To verify the expression levels of these recombinant proteins, *E. coli* BL21 star(DE3) was transformed with the expression vectors and grown in liquid LB medium followed by induction with 0.5 mM IPTG. Figure 2 showed the gel electrophoresis patterns of protein samples visualized by Coomassie Brilliant Blue staining. We noted distinct bands of the expected sizes from crude extracts of the recombinant strains when compared with the control strain BL21/pET28a. SDS-PAGE analysis of the recombinant strain carrying pET-mdlC showed a band corresponding to the molecular weight of 56.4 kDa while strain BL21/pA-yjhGadhP revealed two protein bands (35.4 kDa and 70.0 kDa). The recombinant strain harboring both of the two plasmids gave all three bands. Strain BL21/pA-xylBC revealed the recombinant proteins corresponding to the molecular weights of 26.6 kDa and 31.6 kDa. And the final strain harboring both pE-mdlCxylBC and pA-yjhGadhP overexpressed all of the five enzymes.

### Enhancement of BT production from xylonate by coexpression of the entire pathway

**Figure 1:**
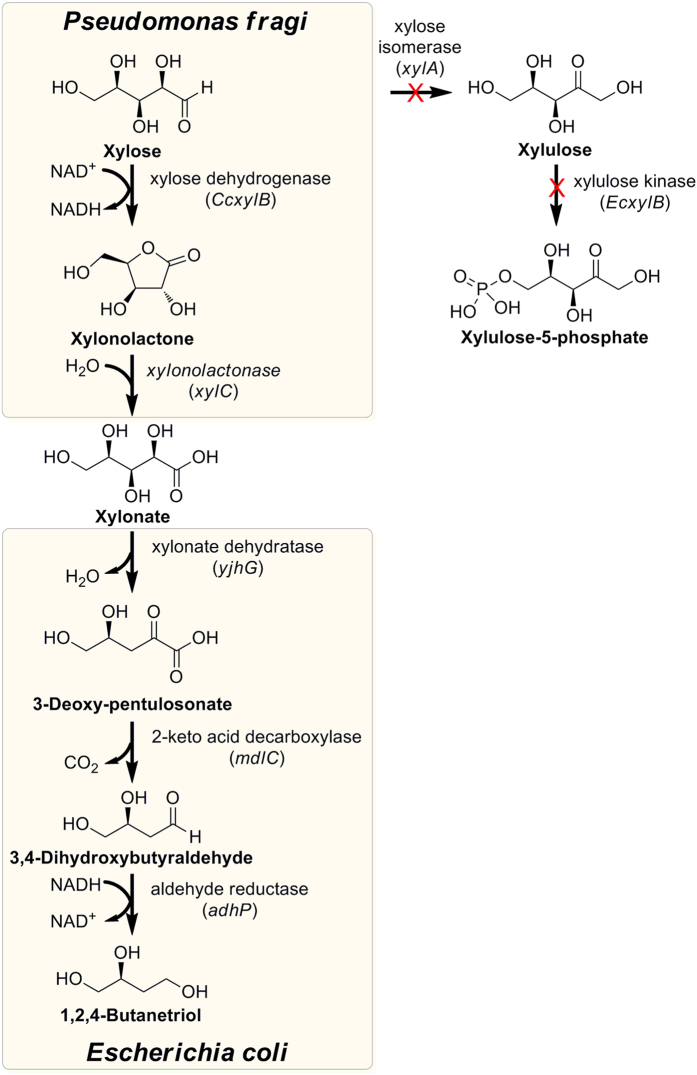
The metabolic pathway from xylose to BT. Xylose is oxidized to xylonolactone by the xylose dehydrogenase (encoded by *CcxylB*). Xylonolactone is hydrolyzed to xylonate by the xylonolactonase (encoded by *xylC*). Xylonate dehydratase (encoded by *yjhG*) catalyzes the dehydration of xylonate to produce 3-deoxy-pentulosonate. Then 3-deoxy-pentulosonate is decarboxylated by 2-keto acid decarboxylase (encoded by *mdlC*) to form 3,4-dihydroxybutyraldehyde. At last, 3,4-dihydroxybutyraldehyde is reduced by the aldehyde reductase (encoded by *adhP*) to generate BT. Xylose isomerase (encoded by *xylA*) and xylulose kinase (encoded by *EcxylB*) responsible for xylose metabolism are knocked out to block the branched pathways.

**Figure 2:**
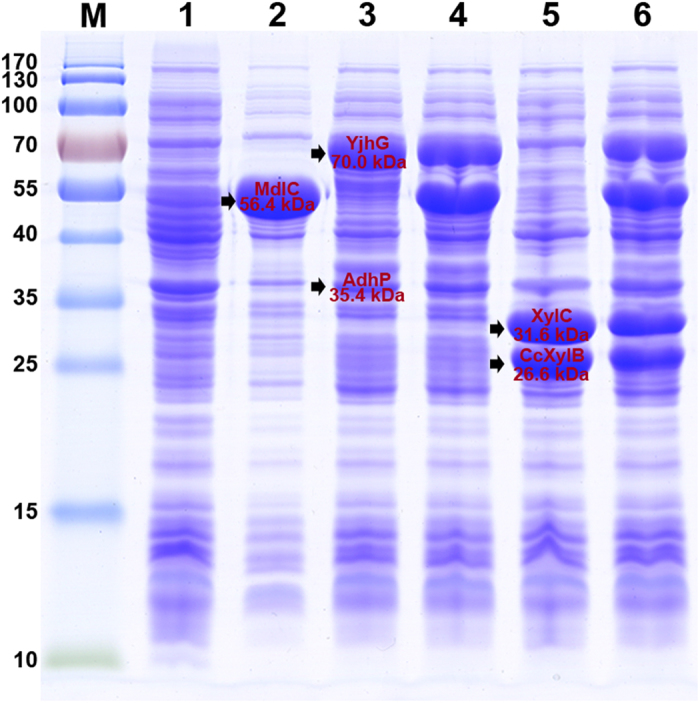
Validation of the expression of different recombinant enzymes in E. coli through SDS-PAGE analysis. Recombinant protein expression was induced using 0.5 mM IPTG for a cultivation time of 4 h at 37 °C. Lane M, prestained protein molecular weight marker; lane 1, crude cells extracts from *E. coli* BL21 star(DE3) harboring pET28a; lane 2, crude cells extracts from strain BL21/pET-mdlC; lane 3, crude cells extracts from strain BL21/pA-adhPyjhG; lane 4, crude cells extracts from strain BL21/pET-mdlC&pA-adhPyjhG; lane 5, crude cells extracts from strain BL21/pA-xylBC; lane 6, crude cells extracts from strain BL21/pE-mdlCxylBC&pA-adhPyjhG. The bands corresponding to the individual proteins were indicated by an arrow.

**Figure 3:**
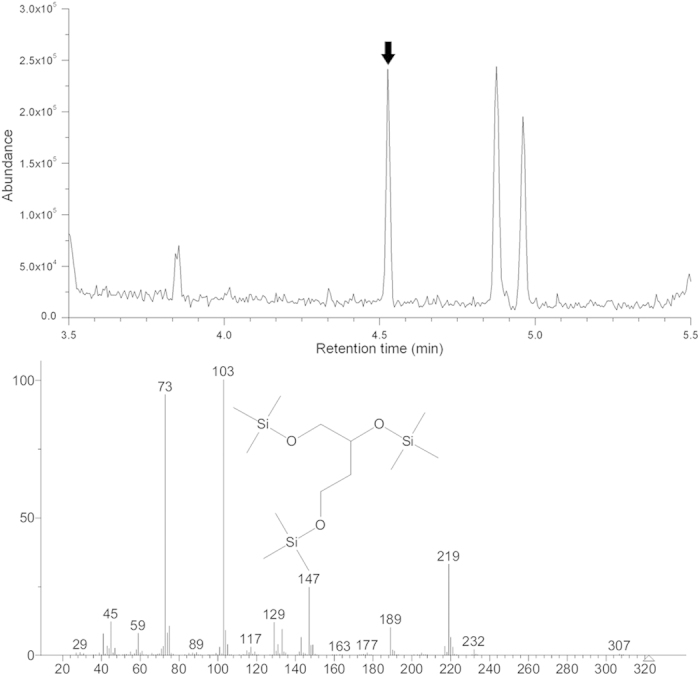
Identification of BT accumulated in the culture broth by GC-MS. (**A**) total ion current (TIC) chromatogram of the silylation products of the fermentation supernatant from *E. coli* BL21 star(DE3) harboring pET-mdlC and pA-adhPyjhG after being induced for 48 h. (**B**) mass spectrum of silylation product of BT (corresponding to the retention time of 4.53 min).

**Figure 4:**
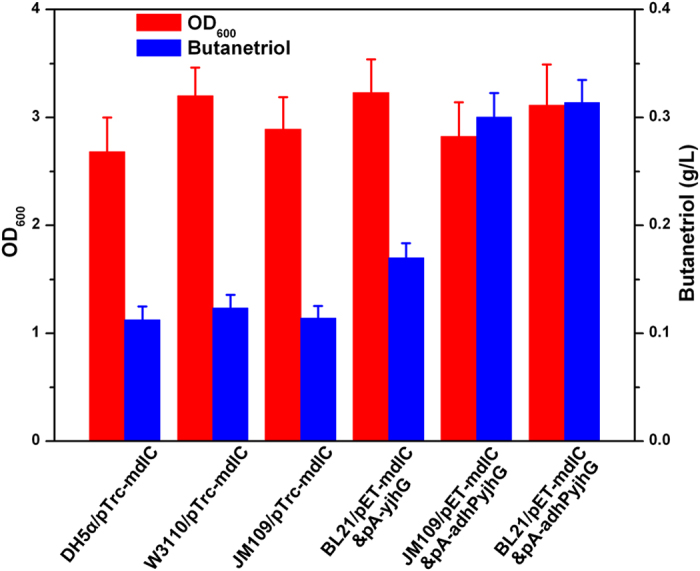
Comparison of BT productions of several different strains. DH5α/pTrc-mdlC, strain DH5α *P. putida* 2-keto acid decarboxylase; BL21/pET-mdlC&pA-yjhG, strain BL21 star(DE3) expressing *P. putida* 2-keto acid decarboxylase and native *E. coli* dehydratase; BL21/pET-mdlC&pA-adhPyjhG, strain BL21 star(DE3) expressing *P. putida* 2-keto acid decarboxylase and native *E. coli* dehydratase and aldehyde reductase. Data were obtained after each strain was induced for 48 h in liquid LB medium supplemented with 5 g/L of potassium xylonate as the substrates for BT production.

It has been demonstrated that heterologous expression of *P. putida mdlC* gene in *E. coli* DH5α led to the synthesis of BT from xylonate^19^. However, this biotransformation process could not be achieved using *E. coli* BL21(DE3) as the host strain. Frost and co-workers have postulated a potential metabolic pathway from xylonate to BT (Fig. 1). Two enzymes, xylonate dehydratase (encoded by *yjhG*) and aldehyde reductase (encoded by *adhP*) are required for the necessary steps of the bioconversion. *E. coli* BL21(DE3) genome does not carry any xylonate dehydratase^25^ and thus cannot metabolize xylonate. To render strain BL21 the ability of producing BT from xylonate, we cloned the *yjhG* and *adhP* genes from strain DH5α into the expression vector pACYCDuet-1 sequentially, resulting pA-yjhG and pA-adhPyjhG. To evaluate the effects of coexpression of the entire pathway on BT production, *E. coli* DH5α, W3110, JM109(DE3) and BL21 star(DE3) was transformed with pTrc-mdlC, pET-mdlC, pA-yjhG, pA-adhPyjhG or the combination of these plasmids. The recombinant strains were cultured using liquid LB medium supplemented with 5 g/L of potassium xylonate under shake-flask conditions. After being induced for 48 h, the bacterial cells were separated by centrifugation and BT produced in the supernatant was derivatized and analyzed by gas chromatography—mass spectrometry (GC-MS). Figure 3 showed the total ion current (TIC) chromatogram and mass spectrum of the silylation products of the fermentation supernatant from strain BL21/pET-mdlC&pA-adhPyjhG. 1,2,4-Tri(trimethylsilyl)butanetriol (corresponding to the retention time of 4.53 min) was identified by matching a standard NIST library. BT production of different engineered strains in the culture supernatant was then directly determined by high-performance liquid chromatography (HPLC). As shown in Fig. 4, cell growth was comparable between these recombinant strains. The titer of BT for strain DH5α/pTrc-mdlC reached 0.11 g/L after 48 h of induction. Similar results were obtained by strain W3110/pTrc-mdlC and JM109/pTrc-mdlC. When the *yjhG* gene was coexpressed with *mdlC*, BT production was enhanced to 0.17 g/L. In the strain JM109/pET-mdlC&pA-adhPyjhG and BL21/pET-mdlC&pA-adhPyjhG coexpressing the entire BT biosynthesis pathway, the final titer of BT was further elevated to 0.31 g/L, which is 1.8-fold higher than the original strain. It has been demonstrated that the aldehyde reductase AdhP could convert aldehydes to alcohols^26^ and reduce the toxicity of the intermediate 3,4-dihydroxybutanal, thus leading to an enhanced production of BT.

### Direct bioconversion of xylose to BT

**Figure 5:**
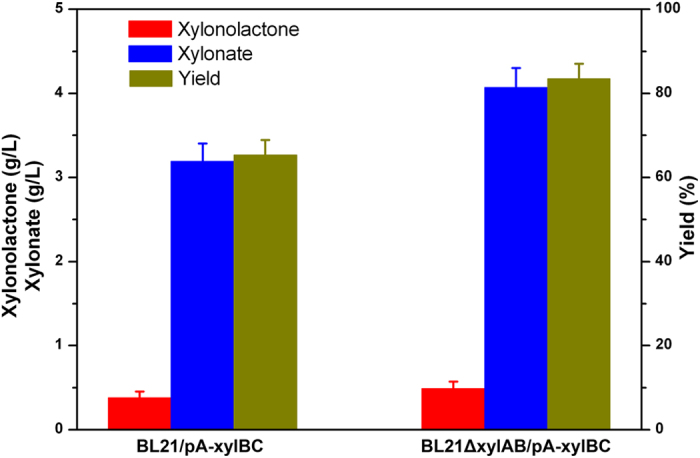
Production of xylonate and xylonolactone through the upstream pathway. BL21/pA-xylBC, strain BL21 star(DE3) expressing *C. crescentus* xylose dehydrogenase and xylonolactonase; BL21ΔxylAB/pA-xylBC, knockout of native *xylA* and *EcxylB* while coexpressing *C. crescentus* xylose dehydrogenase and xylonolactonase. Data were obtained after each strain was induced for 24 h in liquid LB medium supplemented with 5 g/L of xylose.

Previous studies employed a two-step process to synthesize BT from xylose because *E. coli* was incapable of producing xylonate from xylose. The conversion of xylose to xylonate requires two enzymes, xylose dehydrogenase and xylonolactonase. Recently, the xylose dehydrogenase (*CCxylB*) and xylonolactonase (*xylC*) were identified from the freshwater bacterium *C. crescentus*^27^. Here, the two genes were coexpressed in *E. coli* BL21 star(DE3) using the recombinant vector pA-xylBC. Accumulations of xylonolactone and xylonate were detected in the cultures of the recombinant strain. As shown in Fig. 5, 0.38 g/L of xylonolactone and 3.19 g/L of xylonate were produced from 5 g/L of xylose after being induced for 24 h. *E. coli* could utilize xylose via the pentose phosphate pathway for cell growth. The first two genes responsible for xylose catabolism have been recognized as xylose isomerase (encoded by *xylA*) and xylulose kinase (encoded by *EcxylB*)^28^. In order to block the native xylose catabolic pathway, we disrupted the *xylA* and *EcxylB* genes in strain BL21 star(DE3) to create BL21ΔxylAB. The final titers of xylonolactone and xylonate in strain BL21ΔxylAB/pA-xylBC were enhanced to 0.49 g/L and 4.07 g/L, respectively. The molar yield of xylonolactone and xylonate on xylose also reached 83.5%, which is 18.1% higher than strain BL21/pA-xylBC.

In order to integrate the xylonate and BT producing pathway, the *CCxylB* and xylC genes were coexpressed with *mdlC* using the recombinant vector pE-mdlCxylBC. Both pE-mdlCxylBC and pA-adhPyjhG were co-transformed to BL21ΔxylAB competent cells. The resulting strain BL21ΔxylAB/pE-mdlCxylBC&pA-adhPyjhG was grown in LB medium containing 5 g/L of xylose. As expected, accumulation of BT was found in the culture of this engineered strain. BT production after being induced for 48 h reached 0.30 g/L, which is comparable with the strain converting xylonate to BT. The molar yield of BT to xylose reached 8.5%. As xylose, xylonate and many other organic acids shared a similar retention time in the HPLC chromatograph, we use the ion chromatography (IC) to separate these metabolites. IC analysis showed that the initial xylose was completely exhausted at 48 h post-induction, but intermediate metabolites of the BT biosynthesis pathway were detected in the culture broth. This fact indicated that the downstream pathway (xylonate to BT) instead of the upstream pathway (xylose to xylonate) was the rate-limiting step for BT biosynthesis.

### BT production in fed-batch cultivation

**Figure 6:**
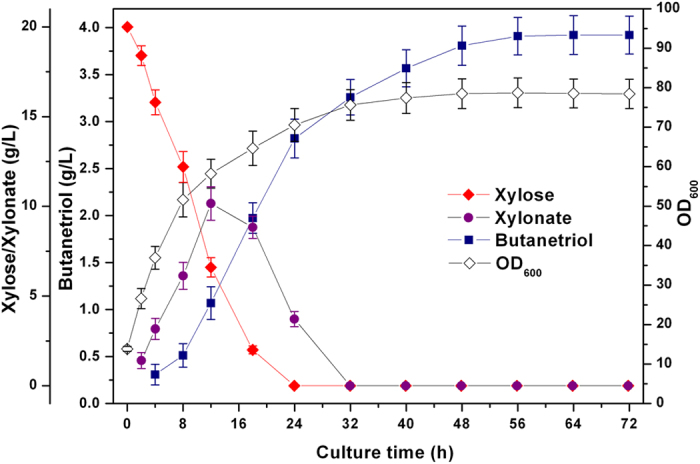
Time profiles for cell density (OD_600_), residual xylose and BT concentrations in the culture broth during fed-batch culture of the engineered strain BL21ΔxylAB/pE-mdlCxylBC&pA-adhPyjhG. Cultures were performed in a 5L laboratory fermentor. 20 g/L of xylose was used as the substrates for BT production. Error bars represent the range of three independent fermentations.

In order to test the suitability of the recombinant *E. coli* strains for larger-scale production of BT, we established fed-batch fermentation based on the results obtained with shake-flask cultures. Strain BL21ΔxylAB/pE-mdlCxylBC&pA-adhPyjhG was cultured in a 5 L-scale laboratory fermenter. Glycerol was selected as the carbon source instead of glucose to avoid catabolite repression of xylose uptake. Cell density, residual xylose and BT accumulation were monitored over the course of the experiment. The residual glycerol concentration was controlled at a low level to avoid acetate formation, which might hamper cell growth and BT detection. Figure 6 showed the time profiles of cell density and concentrations of different metabolites during the whole fermentation period. For approximately 32 h post-induction, the bacteria grew rapidly to an OD_600_ of 75 or so. Most of the xylose was utilized in the first 18 h of fermentation. The intermediate metabolite xylonate reached a maximum titer of 10.2 g/L at 10 h post-induction and was then gradually consumed by the downstream pathway. BT also accumulated rapidly in the culture broth during the first 40 h post-induction and then gradually to a stable value. The highest BT production was obtained after being induced for 56 h, that is, 3.92 g/L, corresponding to a volumetric productivity of 0.89 mg/(L·h·OD_600_). The final molar yield of BT on xylose reached 27.7%. The above results achieved at the fermenter level greatly enhanced the titer of BT in the fermentation broth and demonstrated that this engineered *E. coli* strain had the potential to produce BT in a large scale.

## Discussion

In this work, we integrated the two steps for biotechnological BT production in a single *E. coli* host and the metabolic pathway was further engineered to enhance the carbon flux to BT. Both the titer and yield of BT were enhanced to some extent compared with the original study^19^. These results demonstrated the power of rational design to improve the ability of microorganisms to produce bio-based chemicals. The one-step conversion process also makes the cells maintain redox balance. Reducing equivalent supply is crucial for the biosynthesis of value-added chemicals^29^. The metabolic pathway from xylonate to BT consists of three steps: dehydration, decarboxylation and reduction. One mole of NADH is required for each mole of BT formed. Therefore, the cells should provide additional NADH to facilitate the reduction of 3,4-dihydroxybutanal. The NADH balance forces more carbon flux to be channeled into the TCA cycle, leading to excessive consumption of the carbon sources. The upstream metabolic pathway from xylose to xylonate could produce one mole of NADH for each mole of xylose to be oxidized^30^. NADH consumed by the production of BT could be regenerated through the oxidation of xylose. Therefore, the integration of the upstream pathway and the downstream pathway in a single host could provide appropriate reducing power, which might contribute to BT biosynthesis.

The engineered strain in current study has greatly improved BT production. However, the yield of BT on xylose is still far from the theoretical limits. This result might be due to the following issues. First of all, the rate-determining step for BT biosynthesis is the decarboxylation of 3-deoxy-pentulosonate catalyzed by MdlC. The original activity of this enzyme is a benzoylformate decarboxylase participating in mandelate degradation^20^. 3-Deoxy-pentulosonate is not the natural substrate and thus it shows a much lower substrate specificity and catalytic activity. Therefore, protein engineering of the MdlC decarboxylase to improve its catalytic activity towards 3-deoxy-pentulosonate would be helpful to BT production^31^. On the other hand, there are many competing pathways associated with the BT biosynthesis pathway. For instance, 3-deoxy-pentulosonate can be split to pyruvate and glycolaldehyde^32^ as well as forming 2-amino-4,5-dihydroxypentanoate catalyzed by aminotransferase^33^. The oxidation of 3,4-dihydroxybutanal to 3,4-dihydroxybutyrate might also compete with its reduction to BT. Inactivation of these branched metabolic pathways would be expected to further improve BT production. However, these reactions are always catalyzed by multiple enzymes or key enzymes essential for the cell’s normal metabolism. It is difficult to completely block these branched pathways without affecting cell viability. Therefore, regulating the metabolic flux to BT biosynthesis would be critical to enhance its production.

## Materials and Methods

### Bacterial strains and plasmids construction

A list of bacterial strains and recombinant plasmids was presented in Table 1. Primers used for plasmids construction was provided in Table 2. *E. coli* DH5α was used for gene cloning and *E. coli* BL21 star(DE3) was used as the host for the expression of the recombinant proteins. The chromosomal *xylA* and *EcxylB* genes of strain BL21 star(DE3) responsible for xylose utilization were knocked out using the λ-Red recombination strategy in a previous study, resulting strain BL21ΔxylAB^34^.

The 2-keto acid decarboxylase from *P. putita* (*mdlC*, GenBank accession no.: AY143338) was codon optimized, chemically synthesized and cloned into pET28a or pTrcHis2B vector between *Nco*I and *Eco*RI sites to create pET-mdlC or pTrc-mdlC. The *CCxylB* (GenBank Accession No.: NACL94329) and *xylC* (GenBank Accession No.: NACL94328) genes from *C. crescentus* were also cloned into pACYCDuet-1 vector simultaneously to generate plasmid pA-xylBC in our previous study^34^. Then *CCxylB* was PCR amplified from vector pA-xylBC and cloned into pETDuet-1 vector between *Nde*I and *Kpn*I sites to create pE-xylB. The *mdlC* gene was digested from pET-mdlC using *Nco*I and *Eco*RI, and then cloned into the same restrict sites of pE-xylB, resulting pE-mdlCxylB. The *xylC* gene was amplified from pA-xylBC along with T7 promoter and the PCR product T7xylC was then cloned into pE-mdlCxylB between *Eco*RI and *Not*I sites, to create pE-mdlCxylBC. The coding sequences of native *E. coli yjhG* (Gene ID: 6060334) and *adhP* (Gene ID: 6059208) were generated by PCR using the primers based on the selected sequences around the start or stop codons of the genes and with restriction enzyme sites at 5′ ends. The resulting PCR product of *yjhG* was cloned into pACYCDuet-1 between *Nde*I and *Xho*I sites leading to plasmid pA-yjhG. The *adhP* gene was then cloned into pA-yjhG between *Nco*I and *Eco*RI sites to create pA-adhPyjhG. Successful gene cloning was verified by colony PCR, restriction mapping and direct nucleotide sequencing.

### Protein expression and gel electrophoresis analysis

For the expression of different recombinant proteins, single colonies of *E. coli* BL21 star(DE3) harboring different recombinant plasmids were used to inoculate Luria-Bertani (LB) medium containing appropriate antibiotics and grown at 37 °C overnight. The saturated culture was diluted 1:100 into fresh LB medium and incubated under the same conditions. When the optical density at 600 nm (OD_600_) of the culture reached about 0.6, recombinant protein expression was induced by 0.5 mM isopropyl-β-D-thiogalactopyranoside (IPTG) and growth was continued for 4 h. Cells were collected from 5 ml of bacteria cultures by centrifugation and washed with sterile distilled water. The washed pellets were suspended in 500 μl Tris-HCl buffer (pH 8.0) and subject to ultrasonication. The cell lysates were centrifuged and the supernatant obtained was mixed with 2 × sodium dodecyl sulfate (SDS) sample buffer, heated at 100 °C for 10 min and then analyzed by SDS-polyacrylamide gel electrophoresis (PAGE) according to standard protocols^35^.

### Shake-flask cultivation

To evaluate the BT producing ability of different engineered strains, shake-flask experiments were carried out in triplicate series of 100 ml Erlenmeyer flasks containing 20 ml of liquid LB medium supplemented with appropriate antibiotics. *E. coli* strains were inoculated to the culture medium and incubated in a gyratory shaker incubator at 37 °C and 180 rpm. When the OD_600_ of the culture reached about 0.6, IPTG was added to a final concentration of 0.5 mM to induce the expression of different enzymes. 5 g/L of potassium xylonate or xylose was added to the culture at the same time as the substrates for BT production. Samples were taken at different intervals to determine cell density, residual xylose and BT accumulated in the culture broth during the whole fermentation courses.

### Fed-batch fermentation

For large-scale production of BT, fed-batch cultures were performed in a Biostat B plus MO5L fermentor (Sartorius, Germany) containing 3 L of growth medium (20 g/L tryptone, 10 g/L yeast extract, 5 g/L NaCl and 5 g/L K_2_HPO_4_·3H_2_O) that was sterilized at 121 °C for 20 min. Glycerol (10 g/L), MgSO_4_ (0.12 g/L) and trace elements (1 ml per liter, 3.7 g/L (NH_4_)_6_Mo_7_O_24_·4H_2_O, 2.9 g/L ZnSO_4_·7H_2_O, 24.7 g/L H_3_BO_3_, 2.5 g/L CuSO_4_·5H_2_O, 15.8 g/L MnCl_2_·4H_2_O) were autoclaved or filter-sterilized separately and added prior to initiation of the fermentation. Fermentation was started by inoculating 100 ml of overnight seed culture prepared in LB medium. During the fermentation process, continuous sterile air was supplied at a flow rate of 3 L/min. The temperature was controlled at 37 °C and the pH was controlled at 7.0 via automatic addition of ammonia. Antifoam was added to prevent frothing if necessary. The agitation speed was set at 400 rpm and then associated with the dissolved oxygen (DO) to maintain the DO level above 20% air saturation. Fermentation was first operated in a batch mode until the initial glycerol was nearly exhausted. Then fed-batch mode was commenced by feeding 70% of glycerol at appropriate rates. When the cells were grown to an OD_600_ of about 15, 0.5 mM of IPTG, 20 g/L of xylose and 0.1 g/L of thiamine hydrochloride were added to the culture broth to induce recombinant protein expression and BT production. Samples of the fermentation broth were determined the same as shake-flask cultivation.

### Analytic methods

BT produced in the culture broth was identified using gas chromatography-mass spectrometry (GC-MS). The culture supernatant was mixed with 9 volume of ethanol to deposit proteins and concentrated by reduced pressure distillation. The concentrated sample was then derivatized by bis(trimethylsilyl)trifluoroacetamide (BSTFA): appropriate amounts of the samples were added 1 ml of BSTFA and 0.2 ml of pyridine, and then reacted at 70 °C for 30 min^36^. The reaction mixture was diluted by hexane and then subjected to GC-MS. GC-MS analysis was performed with an Agilent 7890 GC system coupled to a quadrupole rods detector. The GC-MS conditions were as follows: a 30 m HP-5 ms column (internal diameter 0.25 mm, film thickness 0.25 μm); an oven temperature program composed of an initial hold at 100 °C for 2 min, ramping at 10 °C per min to 250 °C, and a final hold at 250 °C for 3 min; an ion source temperature of 220 °C and EI ionization at 70 eV.

BT concentrations in the culture supernatant was determined by an Agilent 1200 series HPLC system equipped with an Aminex HPX-87H (Bio-Rad, Hercules, CA) column (300 × 7.8 mm). All samples were filtered through 0.22 μm syringe filter. Ultrapure water with 5 mM H_2_SO_4_ was used as the eluent at a flow rate of 0.5 ml/min. The oven temperature was maintained at 55 °C. Peaks were detected by a refractive index detector (RID). Quantitation of BT was performed by using the external standard method.

The determination of xylose in the culture broth was performed on an ICS-3000 (Dionex, Sunnyvale, CA) ion chromatography (IC) system. The IC was equipped with an IonPacAS11 anion chromatography column (4.0 mm × 250 mm) and an AG-11 guard column (4.0 mm × 50 mm). Suppression was achieved with anion suppressor (ASRS 300 4 mm). Peaks were detected using electrochemical detector. A mixture of 250 mM NaOH (2%) and H_2_O (98%) was used for elution at a flow rate of 1 ml/min. For sample analysis, another elution step with 80% of 250 mM NaOH was employed to remove the residual components. Data collection and handling were carried out by Dionex Chromeleon software.

## Additional Information

**How to cite this article**: Cao, Y. *et al*. Biotechnological production of 1,2,4-butanetriol: An efficient process to synthesize energetic material precursor from renewable biomass. *Sci. Rep*. **5**, 18149; doi: 10.1038/srep18149 (2015).

## Figures and Tables

**Table 1:** Strains and plasmids used in this study.

Strains or plasmids	Genotype/Description	Source
Strains		
* E. coli* DH5α	*F*^*−*^ *recA endA1 Φ80dlacZΔM15 hsdR17(r*_*K*_^*−*^ *m*_*K*_^*+*^*) λ*^*−*^	Invitrogen
* E. coli* W3110	*F*^*−*^ *λ*^*−*^ *rph-1 INV(rrnD rrnE)*	Coli Genetic Stock Center
* E. coli* JM109 (DE3)	*recA endA1 lacZΔM15 (lac-proAB) hsdR17(r*_*K*_^*−*^ *m*_*K*_^*+*^) IDE3	Promega
* E. coli* BL21 star (DE3)	*F*^*−*^ *ompT hsdS*_*B*_ (r_B_^*−*^ m_B_^*−*^) *gal dcm rne131* (DE3)	Invitrogen
* E. coli* BL21 star (DE3) ΔxylAB	Knockout of *xylA* and *EcxylB* encoding xylose isomerase and xylulose kinase	34
Plasmids		
* *pET28a	*Kan*^*r*^ *oripBR322 lacI*^*q*^ *T7p*	Novagen
* *pTrcHis2B	*Amp*^*r*^ *oripBR322 lacI*^*q*^ *Trcp*	Invitrogen
* *pETDuet-1	*Amp*^*r*^ *oripBR322 lacI*^*q*^ *T7p*	Novagen
* *pACYCDuet-1	*Cm*^*r*^ *oriP15A lacI*^*q*^ *T7p*	Novagen
* *pET-mdlC	pET28a harboring *P. putida mdlC*	This study
* *pTrc-mdlC	pTrcHis2B harboring *P. putida mdlC*	This study
* *pA-xylBC	pACYCDuet-1 harboring *C. crescentus CCxylB* and *xylC*	34
* *pE-xylB	pETDuet-1 harboring *C. crescentus* CC*xylB*	This study
* *pE-mdlCxylB	pETDuet-1 harboring *P. putida mdlC* and *C. crescentus CCxylB*	This study
* *pE-mdlCxylBC	pETDuet-1 harboring *P. putida mdlC* and *C. crescentus CCxylB* and *xylC*	This study
* *pA-yjhG	pACYCDuet-1 harboring *E. coli yjhG*	This study
* *pA-adhPyjhG	pACYCDuet-1 harboring *E. coli yjhG* and *adhP*	This study

**Table 2:** Primers used in this study for plasmids construction or allele verification.

Oligonucleotide primers	Description
xylB_F_NdeI	GGGAATTCCATATGTCCTCAGCCATCTATCCC
xylB_R_KpnI	CGGGGTACCTCAACGCCAGCCGGCGTCGAT
mdlC_F_NcoI	CATGCCATGGCTTCTGTTCACGGTACCACC
mdlC_R_EcoRI	CCGGAATTCTTATTTAACCGGAGAAACGGTAG
T7xylC_F_EcoRI	CCGGAATTCTAATACGACTCACTATAGGGGAATTG
T7xylC_R_NotI	AAGGAAAAAAGCGGCCGCTTAAACCAGACGAACTTCGTGCTG
yjhG_F_NdeI	GGAATTCCATATGTCTGTTCGCAATATTTTTGC
yjhG_R_XhoI	CCGCTCGAGTCAGTTTTTATTCATAAAATCGCG
adhP_F_NcoI	CATGCCATGGGCATGAAGGCTGCAGTTGTTACG
adhP_R_EcoRI	CCGGAATTCTTAGTGACGGAAATCAATCACC
